# Supplementary material for: Harnessing DSB repair to promote efficient homology-dependent and -independent prime editing
Source: Nat Commun. 2022 Mar 24;13:1240. doi: 10.1038/s41467-022-28771-1 (PMC8948305; doi:10.1038/s41467-022-28771-1)
Supplement: Supplementary file 1 — Supplementary Information [file 41467_2022_28771_MOESM1_ESM.pdf]

SUPPLEMENTARY FIGURES

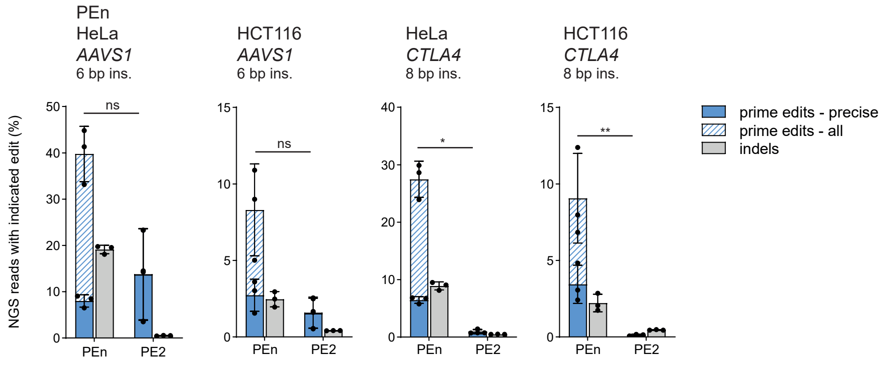


**Supplementary Figure 1 – *Sp*Cas9 nuclease-based prime editing.** NGS analysis of PEn or PE2-mediated targeted DNA insertions of indicated sizes in HeLa and HCT116 cells. Plots show mean ± SD of n=3 biologically independent replicates. “prime edits – all” and “prime edits – precise” categories are superimposed. P-values were determined using Student’s paired t test (two-tailed) * P < 0.05, ** P < 0.01, *** P < 0.001. Calculated P values: HeLa *AAVS1* = 0.3716, HCT116 *AAVS1* = 0.2569, HeLa *CTLA4* = 0.0433, HCT116 *CTLA4* = 0.0028. Source data for Supplementary Figure 1 are provided as a Source Data file.

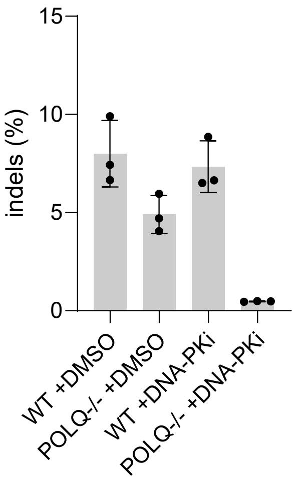


**Supplementary Figure 2 – NHEJ mediates imprecise PEn editing.** NGS analysis of Cas9-induced indels at *AAVS1* in *POLQ-/-* cells with or without DNA-PK inhibition. Plots show mean ± SD of n=3 biologically independent replicates. Source data for Supplementary Figure 2 are provided as a Source Data file.

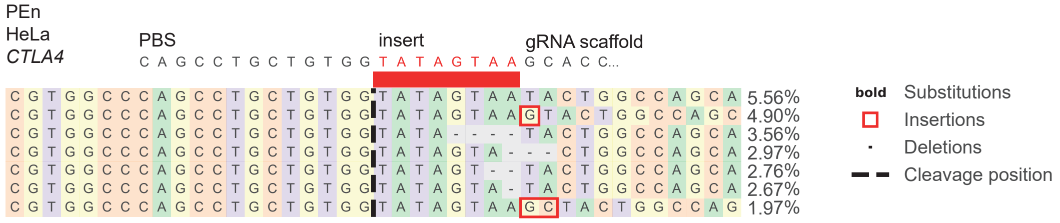


**Supplementary Figure 3**– Representative alignment and frequencies of prime edited alleles of *CTLA4* locus edited with P En using the indicated RT template.

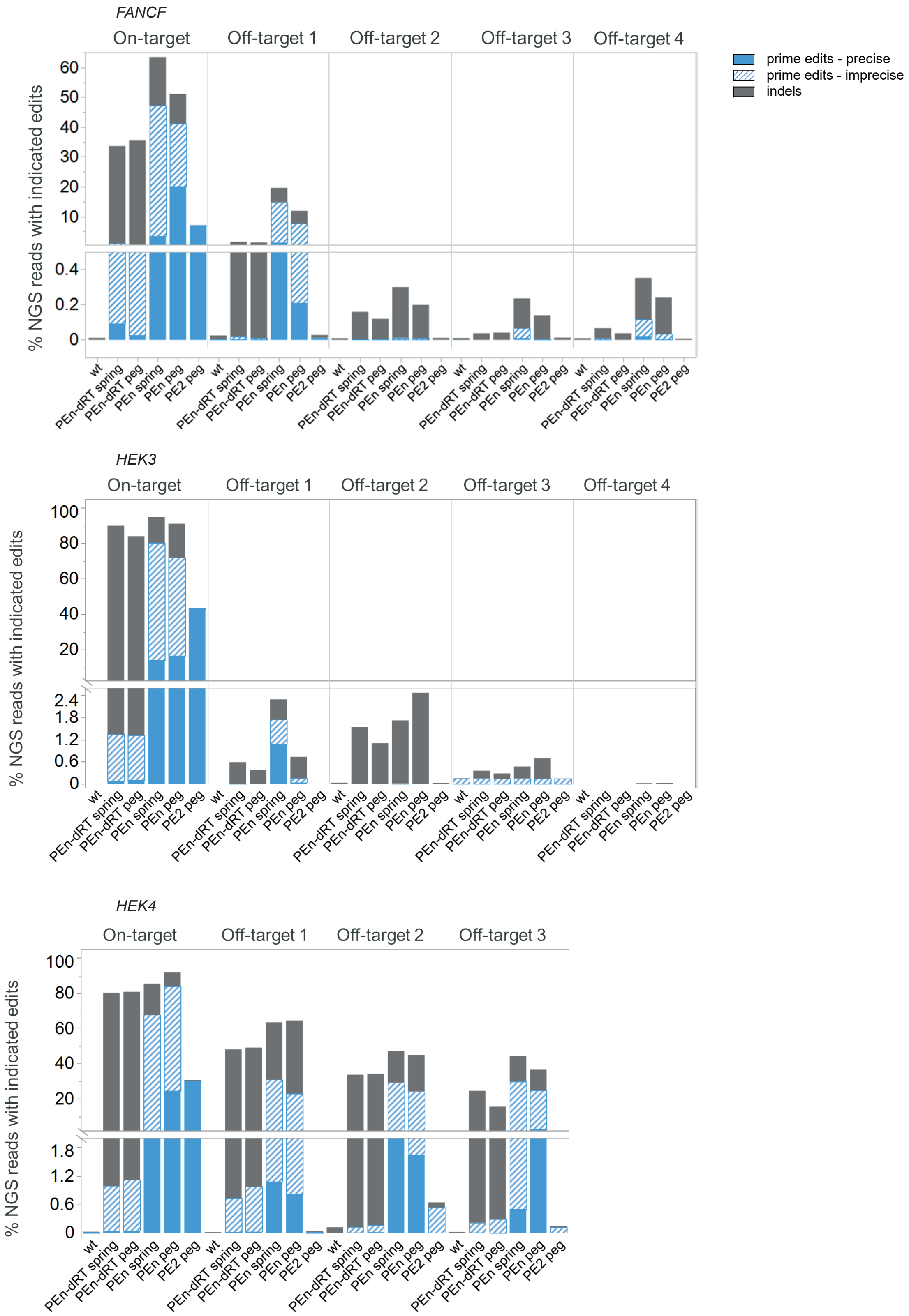


**Supplementary Figure 4 – Off-target analysis of PEn editing.** NGS analysis of editing outcomes at three on-target and eleven off-target sites with indicated editors and peg/springRNAs. Plots show average values of n=3 biologically independent replicates. Indicated editing categories are stacked. Source data for Supplementary Figure 4 are provided as a Source Data file.

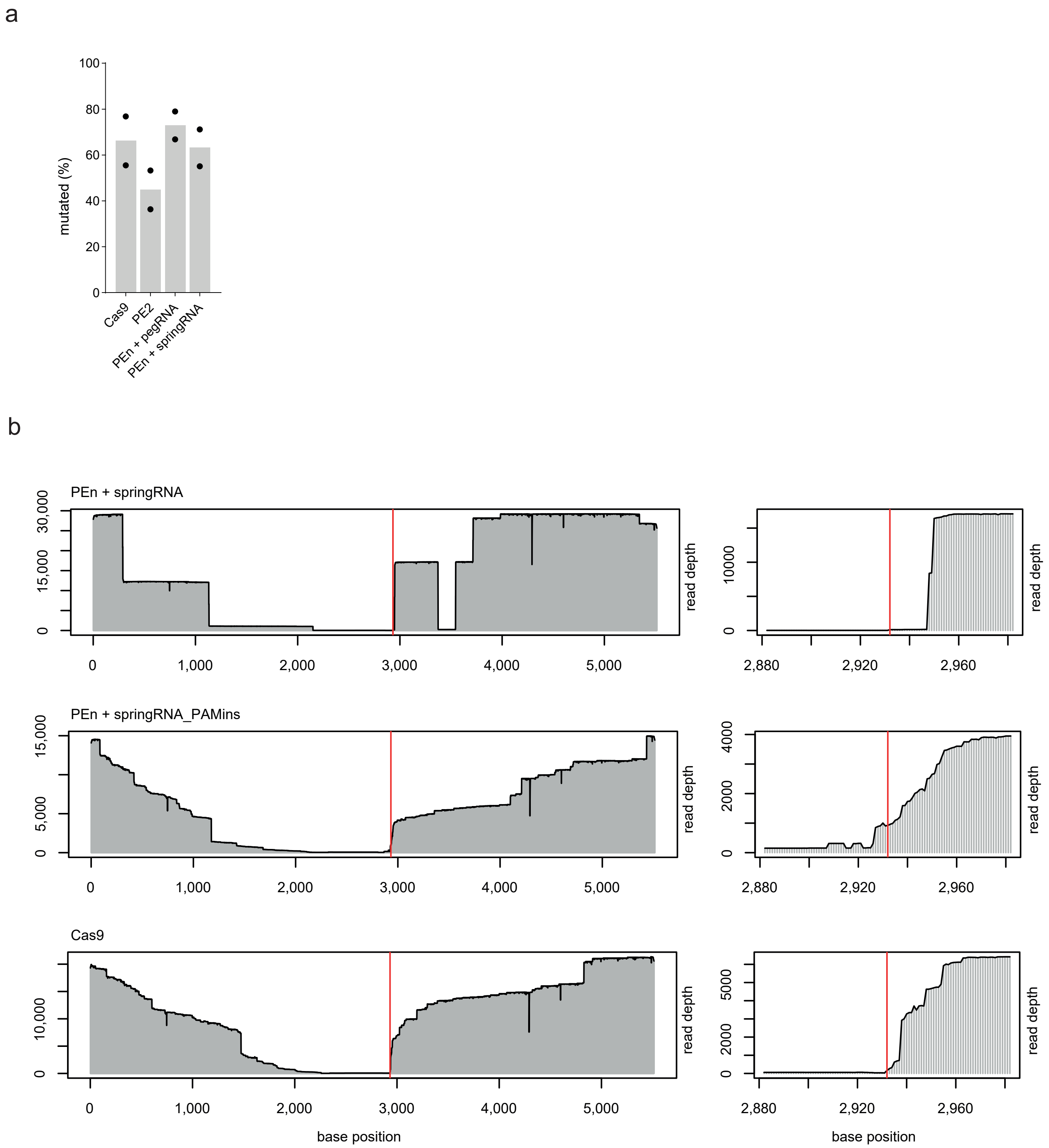


**Supplementary Figure 5 – Large on-target deletion induction by Cas9, PE2 or PEn editing.**

a) NGS analysis of overall editing levels at the *HBEGF* locus in cells edited with Cas9, PE2 and PEn that were used for large deletion analysis. The plot shows mean ± SD of n=2 biologically independent replicates b) Alignment of long *HBEGF* reads from samples targeted with Cas9, PE2 or PEn and harvested after DT selection. Red lines denote Cas9 cleavage site. Source data for Supplementary Figure 5a are provided as a Source Data file.
