# Supplementary material for: Harnessing DSB repair to promote efficient homology-dependent and -independent prime editing
Source: Nat Commun. 2022 Mar 24;13:1240. doi: 10.1038/s41467-022-28771-1 (PMC8948305; doi:10.1038/s41467-022-28771-1)
Supplement: Supplementary file 2 — Reporting Summary [file 41467_2022_28771_MOESM2_ESM.pdf]

nature portfolio

Corresponding author(s): Martin Peterka, Marcello Maresca

Last updated by author(s): Feb 3, 2022

Reporting Summary

Nature Portfolio wishes to improve the reproducibility of the work that we publish. This form provides structure for consistency and transparency in reporting. For further information on Nature Portfolio policies, see our Editorial Policies and the Editorial Policy Checklist.

Statistics

For all statistical analyses, confirm that the following items are present in the figure legend, table legend, main text, or Methods section.

n/a	Confirmed	
☐	☒	The exact sample size (n) for each experimental group/condition, given as a discrete number and unit of measurement
☐	☒	A statement on whether measurements were taken from distinct samples or whether the same sample was measured repeatedly
☐	☒	The statistical test(s) used AND whether they are one- or two-sided<br>*Only common tests should be described solely by name; describe more complex techniques in the Methods section.*
☒	☐	A description of all covariates tested
☒	☐	A description of any assumptions or corrections, such as tests of normality and adjustment for multiple comparisons
☐	☒	A full description of the statistical parameters including central tendency (e.g. means) or other basic estimates (e.g. regression coefficient) AND variation (e.g. standard deviation) or associated estimates of uncertainty (e.g. confidence intervals)
☐	☒	For null hypothesis testing, the test statistic (e.g. F, t, r) with confidence intervals, effect sizes, degrees of freedom and P value noted<br>*Give P values as exact values whenever suitable.*
☒	☐	For Bayesian analysis, information on the choice of priors and Markov chain Monte Carlo settings
☒	☐	For hierarchical and complex designs, identification of the appropriate level for tests and full reporting of outcomes
☒	☐	Estimates of effect sizes (e.g. Cohen's d, Pearson's r), indicating how they were calculated

Our web collection on statistics for biologists contains articles on many of the points above.

Software and code

Policy information about availability of computer code

Data collection	no software was used for data collection
Data analysis	CRISPResso2 (Pinello Lab); JMP 14.1.0 (SAS Institute Inc); GraphPad Prism 9 (GraphPad Software, Inc); minimap2 (Li, 2018); R (3.4.2); python3

For manuscripts utilizing custom algorithms or software that are central to the research but not yet described in published literature, software must be made available to editors and reviewers. We strongly encourage code deposition in a community repository (e.g. GitHub). See the Nature Portfolio guidelines for submitting code & software for further information.

Data

Policy information about availability of data

All manuscripts must include a data availability statement. This statement should provide the following information, where applicable:

- Accession codes, unique identifiers, or web links for publicly available datasets
- A description of any restrictions on data availability
- For clinical datasets or third party data, please ensure that the statement adheres to our policy

The datasets generated during and/or analysed during the current study are available from the corresponding author on reasonable request.

Field-specific reporting

Life sciences study design

All studies must disclose on these points even when the disclosure is negative.

Sample size	No statistical methods were used to predetermine sample sizes. Sample sizes were determined based on literature precedence for genome editing experiments (e.g. Li et al., Nat Comms 2021, https://doi.org/10.1038/s41467-020-20810-z)
Data exclusions	No data excluded.
Replication	Experiments were performed as 2 or 3 biologically independent replicates, as specified for each experiment in the manuscript figure legends
Randomization	Mammalian cells used in this study were grown under identical conditions; no randomization was used.
Blinding	Mammalian cells used in this study were grown under identical conditions; no blinding was used.

Reporting for specific materials, systems and methods

We require information from authors about some types of materials, experimental systems and methods used in many studies. Here, indicate whether each material, system or method listed is relevant to your study. If you are not sure if a list item applies to your research, read the appropriate section before selecting a response.

Materials & experimental systems

n/a	Involved in the study	
☒	☐	Antibodies
☐	☒	Eukaryotic cell lines
☒	☐	Palaeontology and archaeology
☒	☐	Animals and other organisms
☒	☐	Human research participants
☒	☐	Clinical data
☒	☐	Dual use research of concern

Methods

n/a	Involved in the study	
☒	☐	ChIP-seq
☒	☐	Flow cytometry
☒	☐	MRI-based neuroimaging

Eukaryotic cell lines

Policy information about cell lines

Cell line source(s)	HEK293T (ATCC CRL-3216), HEK293T POLQ-/- (Synthego CRISPR KO pool), HCT116 (ATCC CCL-247), HeLa (ATCC CCL-2)
Authentication	Cells were authenticated by the supplier using STR analysis.
Mycoplasma contamination	all cell lines tested negative for mycoplasma
Commonly misidentified lines (See ICLAC register)	no commonly misidentified cell lines were used in this study
