# Supplementary material for: Harnessing DSB repair to promote efficient homology-dependent and -independent prime editing
Source: Nat Commun. 2022 Mar 24;13:1240. doi: 10.1038/s41467-022-28771-1 (PMC8948305; doi:10.1038/s41467-022-28771-1)
Supplement: Supplementary file 3 — Description of Additional Supplementary Files [file 41467_2022_28771_MOESM3_ESM.pdf]

Title: Supplementary Data 1

Description: a) List of gRNA sequences used in this study b) list of primers used to generate amplicon sequencing libraries c) list of input parameters for CRISPResso analysis
